# A case report of a very late response to 5-azacytidine in a patient with lower risk myelodysplastic syndrome: Time to update treatment guidelines for lower risk patients

**DOI:** 10.1097/MD.0000000000010505

**Published:** 2018-04-27

**Authors:** Nikolaos Spetsieris, Panagiotis Diamantopoulos, Konstantinos Zervakis, Nefeli Giannakopoulou, Niki Rougala, Georgios Garefalakis, Vasiliki Skarlatou, Nora-Athina Viniou

**Affiliations:** National and Kapodistrian University of Athens, Laikon General Hospital, 1st Department of Internal Medicine, Hematology Unit, Greece.

**Keywords:** 5-azacytidine, International Prognostic Scoring System, late response, myelodysplastic syndromes, prognostic system

## Abstract

**Rationale:**

The hypomethylating agent 5-azacytidine has been approved in Europe for patients with intermediate 2 and high (i.e., higher) risk myelodysplastic syndrome according to the International Prognostic Scoring System (IPSS). A total of 91% of all first responses in higher risk patients occur within 6 cycles of treatment; however, data regarding the time to first response in clinical trials with lower risk patients are not available.

**Patient concerns:**

Our case describes the late response of a lower risk (intermediate 1 according to the IPSS and intermediate according to the IPSS-R) patient to 5-azacytidine treatment.

**Diagnosis and interventions**: Once diagnosed, the patient started supportive treatment due to persistent pancytopenia and recurrent infections. The use of a hypomethylating agent was decided because the patient was transfusion dependent, and suffering from recurrent severe febrile infections due to neutropenia. Other possible causes of fever except infections in the context of his neutropenia were excluded.

**Outcomes**: After the 12th cycle of 5-azacytidine the patient showed a hematologic response, with transfusion independency and with no recurrent febrile episodes.

**Lessons:**

This case report probably indicates that a subset of patients who belong to the lower risk category according to the previous prognostic systems and to the intermediate one according to the IPSS-R, may benefit from prolonged treatment with the drug. The indication of 5-azacytidine in Europe for patients with higher risk myelodysplastic syndrome (MDS) (according to the IPSS) could possibly include a wider range of patients if updated according to the IPSS-R.

## 1 Introduction

Myelodysplastic syndromes (MDSs) are a wide group of hematopoietic disorders affecting mainly older patients, with cytopenias and possible transformation into acute myeloid leukemia being the major clinical problems.^[1]^ Patients are stratified according to their prognosis and therapeutic decisions are based upon the prognostic category.^[1]^ They are schematically separated into higher risk (corresponding to IPSS high or intermediate 2 risk) and lower risk (corresponding to IPSS low or intermediate 1 risk).^[2]^ The hypomethylating agent 5-azacytidine has been approved in Europe for patients with intermediate 2 and high risk myelodysplastic syndrome according to the International Prognostic Scoring System (IPSS).^[3]^ A total of 91% of all first responses in higher risk patients under treatment with 5-azacytidine occur within 6 cycles of treatment^[4,5]^; however, data regarding the time to first response in clinical trials with lower risk patients are not available. Our case describes the late response of a lower risk patient (intermediate 1 according to the IPSS or intermediate according to the IPSS-R) to 5-azacytidine treatment. The need to update the studies including lower risk patients under treatment with 5-azacytidine is outlined, according to the IPSS-R. The drug's indication should probably include a group of patients that otherwise cannot receive treatment with 5-azacytidine, and lose the opportunity to benefit from it.

## 2 Case presentation

A 66-year-old male patient was referred to the hematology department due to persistent leukopenia and recurrent febrile episodes. The patient was a smoker, with a history of a transurethral prostatectomy due to benign prostatic hyperplasia 2 years ago and a myelodysplastic syndrome diagnosed one year ago. Upon admission, the general clinical examination showed only severe pallor; the rest was unremarkable. The laboratory results revealed grade 2 anemia, neutropenia and thrombocytopenia.

A bone marrow biopsy showed primary myelodysplastic syndrome with a low percentage of blasts (4%), which was categorized according to the WHO 2008 classification ^[6]^ as refractory cytopenia with multilineage dysplasia (RCMD). Cytogenetic testing revealed a normal karyotype. The patient was categorized as intermediate risk according to the IPSS-R (International Prognostic Scoring System) and intermediate 1 according to the IPSS.

Due to persistent pancytopenia and recurrent infections, the patient started supportive treatment with granulocyte colony stimulating factor (GCSF), an erythropoiesis stimulating agent (epoetin- alfa) and danazol. Ηe also received 25 units of packed red blood cells in 8 months, and he was often admitted with recurrent febrile episodes because of severe infections including *Escherichia coli* septicemia and *Clostridium difficile* colitis. The patient had severe weight loss during this period (his body mass index was 18.5), with a high MUST (Malnutrition Universal Screening Tool) score (4) and was stratified as high risk for malnutrition. His ECOG Performance Status had become 2. In order to exclude any possible causes of fever, except infections due to his neutropenia, laboratory tests were performed for rheumatological disorders (Antinuclear Antibodies, c and p—antineutrophil cytoplasmic antibodies, anti-Jo1, rheumatoid factor), sarcoidosis (Serum Angiotensin Converting Enzyme), and specific infections (Wright abortus, suis and melitensis, full hepatitis panel, human immunodeficiency virus antibodies, cytomegalovirus IgM antibodies, Ebstein Barr virus IgM antibodies, qualitative polymerase chain reaction for cytomegalovirus detection in blood, and anti-Leishmania antibodies), which were all negative. Gluten enteropathy antibody testing was performed, with measurement of serum antiendomysial IgG antibodies, antideaminated gliadin peptide IgG and IgA antibodies, antitissue transglutaminase IgG and IgA antibodies, all of which were within normal ranges. Testing for immunological deficiency and primary hypogammaglobulinemia were negative and complement levels were normal. Immunophenotyical analysis of lymphocyte subpopulations in the peripheral blood showed a decrease in the absolute count of T lymphocytes, a significant decrease in the percentage and absolute count of B lymphocytes as well as NK lymphocytes. Flow cytometry analysis of a bone marrow aspirate revealed a normal percentage of lymphocyte subpopulations in the bone marrow. Thyroid function testing (thyroid stimulating hormone, T3, T4) was normal and antithyroid antibodies (antithyroglobulin antibodies, antimicrosomial antibodies) were within normal range. Tumor markers (carcinoembryonic antigen, carbohydrate antigen 19-9, α-fetoprotein) were within normal range. Furthermore, the patient was evaluated with upper and lower gastrointestinal endoscopy. Oesophaogogastroduodenoscopy findings were consistent with a grade 1 chronic oesophagitis, chronic reactive gastritis, duodenitis with partial villous atrophy, crypt hyperplasia, with a mild histological malabsorption syndrome (grade 1), possibly an incomplete expression of gluten enteropathy. Colonoscopy revealed polyps of the large intestine which were endoscopically removed and histologically evaluated as benign (B12, folate).

The use of a hypomethylating agent was decided because the patient was transfusion dependent, without a significant improvement of his hematocrit and suffering from recurrent severe infections due to neutropenia. The patient started treatment with subcutaneous 5-azacytidine at 75 mg/m^2^/day for 7 days in 28-day cycles. The patient was regularly transfused and supported with GCSF. After the 12th cycle the patient showed a hematologic response, with transfusion independency and with no recurrent febrile episodes. He is currently receiving the 16th cycle and remains in remission, requiring no red blood cell transfusions or granulocyte colony stimulating factors, with a platelet count finally within normal range. Anemia persisted until the 12th cycle and the patient continued transfusions. After the 12th cycle his hemoglobin levels were stable at around 11.6 g/dL, and he became transfusion independent. There were no infectious complications after the third cycle of treatment.

## 3 Discussion

The most significant characteristics of myelodysplastic syndrome (MDS) are morphologic dysplasia and impaired maturation of hematopoietic cells, which lead to insufficient hematopoiesis and consequently to peripheral blood cytopenias.^[7]^ Prognosis of the disease determines treatment selection, therefore the use of an accurate prognosis assessment score system is important. The International Prognostic Scoring System (IPSS) for MDS was proposed in 1997 and revised in 2012 (IPSS-R). Several other relevant scoring systems have been proposed based on patient studies, as the WHO prognostic scoring system (WPS), the global MD Anderson score, and the French prognostic scoring system.^[7]^ Factors taken into account in the IPSS system include cytogenetics, bone marrow blast percentage, and depth of cytopenias, in order to separate patients into categories representing their risk for AML transformation and their expected overall survival.^[1]^

The only curative treatment option for MDS is allogeneic hematopoietic stem cell transplantation, which is performed in fit patients with high-risk disease. Currently, 3 agents have been approved for the treatment of MDS: 5-azacytidine, decitabine, and lenalinomide.^[8]^

The antineoplastic effects of 5-azacytidine are based on cytotoxicity on abnormal hematopoietic cells in the bone marrow and hypomethylation of the DNA. The drug has minimal effect on nonproliferating cells, as it is incorporated in the DNA during the S phase of the cell cycle. For maximal demethylation to occur, prolonged exposure to the drug is necessary. Furthermore, it has been shown that despite the persistence of the neoplastic cytogenetic clone, or the emergence of new ones, continued exposure to 5-azacytidine leads to an improved overall survival.^[9,10]^ The drug may affect the differentiation and proliferation of the abnormal clone without always leading to complete remission, suggesting that prolonged administration leads to not only the initial effect but also to an enhancement of the drug's action.^[9]^

The median time to any first response in the AZA-001 trial (which included intermediate 2 and high-risk patients) was estimated at 2 cycles, with a range of 1 to 16 cycles. 91% of all first responses occurred within 6 cycles of treatment, while the remaining 9% of the patients continued treatment and achieved first response by 12 cycles except for 1 patient that responded at cycle 16. The first response was also the best for 52% of the patients, while 48% achieved a better response with continued administration of 5-azacytidine.^[4]^ In an analysis of 3 CALGB trials (8421, 8921, 9221) with 5-azacytidine for the treatment of MDS, the median number of cycles to first response was 3, with a range of 1 to 17 cycles, and 90% of responses were seen by cycle 6.^[5]^ A response was achieved by 75% of the responders by cycle 4 and the other 25% responded later.^[5]^ The trials mentioned above describe the response of patients with intermediate 2 and high risk myelodysplastic syndrome per the IPSS.

The effectiveness of 5-azacytidine in low- and intermediate-risk myelodysplastic syndrome has been evaluated, showing a survival advantage compared to patients who received best supportive care. In the CALGB 9221 trial, which included patients from all IPSS risk groups, median time to first response was 64 days (5-azacytidine was administered in 28-day cycles).^[11]^ In a randomized phase 2 trial of 5-azacytidine +/− epoetin-beta in low or intermediate 1 risk according to the IPSS myelodysplastic syndromes resistant to erythropoiesis stimulating agents, the overall response rate after 6 cycles of 5-azacytidine was 34,7%.^[12]^ In a study evaluating the response to three alternative dosing schedules of 5-azacytidine in patients with myelodysplastic syndrome, in which 63% were lower risk according to the French- American- British classification system (refractory anemia, refractory anemia with ringed sideroblasts, chronic myelomonocytic anemia with <5% bone marrow blasts), for those who achieved hematologic improvement, onset occurred during the first two cycles for 82%, 56%, and 90% of patients in the AZA 5–2–2, AZA 5–2–5, and AZA 5 groups, respectively.^[13]^ In a retrospective study of 74 patients enrolled in an Italian named patient program evaluating the efficacy of 5-azacytidine in the treatment of low risk (according the IPSS) myelodysplastic syndrome, 77% of the responses occurred within the first 6 cycles and 59% achieved their best response between the 4th and 6th cycle. The remaining 23% of the responses were observed after the 6th cycle.^[14]^

Although data regarding time to first response and time to best response was available from studies with higher risk patients,^[4,5]^ we could not find the time range of responses in lower risk patients according to IPSS treated with 5-azacytidine, after reviewing the literature. Our case describes a patient with an intermediate risk according to IPSS-R and intermediate 1 according to IPSS myelodysplastic syndrome demonstrating a late response to 5-azacytidine therapy after the 12th cycle. It has been mentioned that prolonged exposure to the drug improves overall survival, possibly due its maximal hypomethylation activity.^[9]^ However, such a prolonged treatment with a late response has not been previously reported in patients with lower risk disease according to the IPSS. The special characteristics of the disease of this subset of patients which show late response to 5-azacytidine must be defined, in order to discern the patients that probably require prolonged treatment and may eventually benefit in the long term despite failure to demonstrate a response in the median time frame.

Our case probably indicates that a subset of patients who belong to the lower risk category according to the previous prognostic systems and to the intermediate according to the IPSS-R, may benefit from prolonged treatment with 5-azacytidine. In all the previous studies with 5-azacytidine, including AZA 001 and CALGB 9221, patient risk stratification was based on the IPSS prognostic scoring system.^[4,5,11–14]^ Through the application of the IPSS-R to the studies’ patient data, new conclusions could be drawn regarding the effectiveness of 5-azacytidine in the lower risk group. Patients in the intermediate risk subgroup per the IPSS-R, who could benefit from 5-azacytidine treatment, would be stratified as intermediate 1 risk by the IPSS and thus would be excluded from trials such as AZA-001. Furthermore, the indication of 5-azacytidine in Europe referring to patients with intermediate 2 and high risk MDS (according to the IPSS) could possibly include a wider range of patients if updated according to the IPSS-R. Therefore, a meta-analysis of the previous studies which include low risk patients using the new prognostic system IPSS-R is probably needed.

## Author contributions

**Conceptualization:** Nikolaos Spetsieris, Panagiotis Diamantopoulos, Nora-Athina Viniou.

**Data Curation:** Nikolaos Spetsieris, Panagiotis Diamantopoulos, Konstantinos Zervakis, Nefeli Giannakopoulou, Niki Rougala, Georgios Garefalakis, Vasiliki Skarlatou, Nora-Athina Viniou.

**Formal Analysis:** Nikolaos Spetsieris, Panagiotis Diamantopoulos, Nora-Athina Viniou.

**Investigation:** Nikolaos Spetsieris.

**Methodology:** Nikolaos Spetsieris, Panagiotis Diamantopoulos, Nora-Athina Viniou.

**Supervision:** Nikolaos Spetsieris, Panagiotis Diamantopoulos, Nora-Athina Viniou.

**Validation:** Nikolaos Spetsieris, Panagiotis Diamantopoulos, Nora-Athina Viniou.

**Writing–original draft:** Nikolaos Spetsieris, Panagiotis Diamantopoulos, Nora-Athina Viniou.

**Writing–review & editing:** Panagiotis Diamantopoulos, Nora-Athina Viniou.
